# The Cause and Prevention of Rape*Read before Section on Jurisprudence, at the Thirty-sixth Annual Meeting, State Medical Association, Austin, Texas, April 27, 1904.

**Published:** 1904-05

**Authors:** F. E. Daniel

**Affiliations:** Austin, Texas, President State Medical Association of Texas; First Vice-President International Congress on Tuberculosis 1904; Editor Texas Medical Journal, Etc.


### The Cause and Prevention of Rape.*

BY F. E. DANIEL, M. D., AUSTIN, TEXAS,

President State Medical Association of Texas; First Vice-President International Congress on Tuberculosis 1904; Editor Texas Medical Journal, Etc.

**Sadism in the Negro.**

That attribute of our nature we call "love," and which, with civilized people, is the tie that binds the family and society, is the highest, noblest and most beautiful that actuates man. It is the mainspring of human ambition and endeavor. Upon it are founded marriage, home, the family and social order. It prompts to the

*Read before Section on Jurisprudence, at the Thirty-sixth Annual Meeting, State Medical Association, Austin, Texas, April 27, 1904.

acquisition of fame and fortune, the founding of the home, and the rearing of a family, the upbuilding of society and civilization. Its physical and psychic basis is that instinct which God has implanted in every animal creature, from the moneron to man, to insure a perpetuation of the species. Around this powerful and compelling impulse sentiment has woven the most beautiful and exalting emotions of the heart, sympathy, friendship, affection, pity, mercy and charity. It has been the theme of the poet in all ages. Stripped of these sentiments it stands revealed in the hideous form of lust,—animal desire, brute passion, as primitive man and his progenitors knew it,—like a rugged rock, around which, to conceal its monstrous form, nature has woven the tendrils and foliage and flowers of a beautiful clinging creeper. Divested of these refined and refining features, and unrestrained by the many inhibitions which society, law, religion and ethics have placed upon it, it is a power for evil,—an unchained demon, a cyclone, an irresistable force which, in the history of the world and the evolution of man, has wrought untold woe and misery. I quote from that rare and curious book, Burton's *Anatomy of Melancholy* (1652). The author treats of this fundamental instinct as "Heroic Love," and says (p. 448):

"Love indeed, first united provinces, built cities, and by a perpetual generation, makes and preserves mankind, propagates the church; but if it rage, it is no more love, but burning lust, a disease, frenzy, madness, hell; * * * a vehement purturbation of the mind, a monster of nature. * * * It subverts kingdoms, overthrows cities, towns, families; mars, corrupts and makes a massacre of men. Thunder and lightning, wars, fires, plagues, have not done that mischief to mankind, as this burning lust, this brutish passion. Let Sodom and Gomorrah, Rome, Troy and Corinth, and I know not how many cities bear record, * * * and all succeeding ages will subscribe; * * * all histories are full of these basilisks. Besides those daily murders, effusion of blood, rapes, riots and immoderate expense to satisfy their lusts, bigamy, shame, loss, torture, punishment, disgrace, loathsome disease that proceeds from thence worse than pestilent fevers, torments the body, and that feral melancholy which crucifies the soul in this life and damns to everlasting torments in the world to come. Notwithstanding they know these and many such miseries, threats, tortures, will come upon them, * * * yet out of * * * a depraved nature, or love's tyranny, which so furiously rageth * * * they go down headlong to their own perdition; * * *

they will commit folly with beasts, men, 'leaving the natural use of women,' as Paul sayeth (Romans 1:27), 'burned in lust one toward another, and man with man wrought filthiness.' * * * Solomon's wisdom was extinguished in this fire of lust. Samson's strength enervated, piety in Lot's daughters quite forgotten, gravity of priesthood in Eli's sons, reverend old age in the elders that would violate Susanna, filial duty in Absolom to his stepmother, brotherly love in Ammon toward his sister. Human,—divine laws,—precepts, exhortations, fear of God and man,—fair, foul means,—fame, fortune, shame, disgrace, honor, can not oppose, stave off or withstand the fury of it."

This instinct, being fundamental in man, as in all animals, and the most powerful, is, as would naturally be expected, subject to many and extreme aberrations; and these, being the expression of a disordered psychic state, have led, in all ages, to the gratification of the desire by the most unnatural and revolting methods and practices.

It is only within recent years that these aberrations have been studied from a scientific standpoint, and their forensic importance, and the psychic relations they bear to other instincts and emotions pointed out, and a rational therapeusis suggested. It would be out of place here, and it is unnecessary, to enter into an analysis of them. Havelock-Ellis, Schrenck-Notzing, Kraft-Ebing, Moll, Kiernan and others have done that, and in their studies have revealed that they are psychopathologic, and come, often, properly within the domain of insanity. The revelations in these classical works will stagger the credulity of any but a student of sex psychology and psychopathology; much of it is so revolting that the authors feel obliged, in order to partly palliate the weakness and baseness of man, and not disgust the uninformed or chance reader, to veil many details in terms of the learned languages.

That there is a psychic relation, for instance, between lust and murder, between lust and cruelty, between lust and the religious emotion, between lust and music and art, is a fact, startling within itself. Further, that these emotions are in certain psychopathic organisms, inter-convertable, like the various forms of energy in the domain of physics, or co-existent in the same individual, and one may stand for the equivalent of the other; that the libido may find satisfaction, vicariously, at least to some extent, in the expression, gratification and vent of the associated emotion or impulse, is well established. It is more than a coincidence that most religious conversions occur about the time of puberty in both sexes, when the sex nature is developing and is strong, and repression necessary and enforced. We have to accept this psychic relation as a fact, without attempting an explanation of it. For that see the works mentioned; also Spencer (*Principles of Sociology*), Sanger (*History of Prostitution*), Ferri (*Criminal Anthropology*), etc. No fact is better established than that the infliction of pain—especially spanking, or flagellation on the nates, is a powerful excitant to sexual desire, a fact that is not known, but which should be known to teachers and parents who inflict corporal punishment on children. It is demonstrably true that in some boys the first excitation of the sexual impulse is caused by spanking, even in very young children, —and they are thus incited to masturbation (Kraft-Ebing.) The phychological relation of pain to sexual desire, and to the religious emotion, is illustrated in the practices of a religious sect in Southern Europe, known as the Flagellantes. It is a religious ceremony still practiced in France and other parts of Europe, for the devotees to pass in procession through the streets, stripped to the waist, each one armed with a whip with which he lashes the bare back of the one preceding him or her. Sanger says these emotional outbursts lead to great sexual excitement, and terminate in the indulgence of sexual excesses. Not only does the infliction of pain by the lash provoke the sexual impulse in the sufferer, but in some individuals it also powerfully arouses the libido in him or her who inflicts it, or even witnesses it. It increases the desire and intensifies the pleasure at the crisis; and it is for this abominable reason, Ellis says, that certain teachers (psychopathic), flog boys on their bare nates; and both Ellis, Kraft-Ebing and Spencer tell us of an infamous "Judge Jeffries," an English magistrate, who habitually had women prisoners stripped and whipped, because it gave him sexual pleasure. In some of these unfortunate perverts, the sight of blood alone, will both excite and satisfy the sexual desire; and in others is indispensable to satisfactory coitus.

It would seem that depravity and perverted sexual instinct could go no further; but Ellis, Kierman and others point out a psychological relation between the libido and hunger, which, in extreme cases, leads to anthropophagy. That "Jack, the Ripper," was of this class is now generally believed, as well, perhaps, as was that mysterious murderer of young negro girls in Austin some years ago, in as much as in their victims no evidence of rapê was found; but in the former case certain parts of the sexual system of the women were missing. Of this class, also, doubtless are those unfortunates who can have sexual pleasure only when they perform on their partners, the function attributed to the vampire. Sanger gives us an account of a courtesan, famous (or infamous) in history, who became the type of this class of perverts. He says: "The most famous of all the *auletrides* (prostitutes; the flute players of ancient Athens), was Lamia, who, after being the delight of Alexandria and of King Ptolemy for some fifteen or twenty years, was taken, with the city, by Demetrius of Macedon, and raised to the rank of his mistress. She was forty years of age at this time, yet her skill was such that she ruled despotically her dissolute lover, and left a memorable name in Greek history. The ancients asserted that she owned her name Lamia, which means a sort of vampire or blood-sucker, to the most loathsome depravities." (See also Plutarch's Life of Demetrius.)

Tiberius and Nero, the two most infamous of all the infamous Cæsars, are striking historical types of the lust murderer. They ravished and killed scores of very young girls, preserving the heads of the most beautiful, as we are informed by Sanger, and other authors.

This horrible form of sexual perversion is known as "sadism." Dunglison and Gould define it to be, "sexual perversion which leads to rape, with circumstances of great violence and inhuman cruelty." A certain Marquis de Sade, who lived at the time of the French Revolution, was a modern type of the love and pain pervert. He devoted his time and prostituted his remarkable talents to writing the most revolting books, descriptive of his experiences in the gratification of his depraved sexual propensities by the most horrible and unnatural methods, descriptions doubtless colored, somewhat by his imagination, as he wrote much of it while in prison. Science has bestowed his name upon that distressing form of sexual perversion. His case furnishes an illustration, too, of the fact, almost paradoxical, that such perversions are not confined to the naturally brutal, the coarse and vulgar, but may be conjoined to a highly intellectual and ethical nature, for excepting sexual morals, his was both. He did much charity, and saved friends from the guillotine. It is to the credit of Napoleon that he caused all these and similar libidinous books to be burned. A popular knowledge of this and other forms of perverted sexuality and abominable practices long anterated Sade, however. Many instances of sadism are to be found throughout human history—from the earliest recorded times. Moses legislated against certain sexual crimes prevalent in his day and known even to our jurisprudence. (See the 18th chapter of Leviticus.)

Ellis says the key to the psychic relation of love to pain and the monstrous acts of certain depraved men, is found in animal courtship, meaning the combats between males for the possession of the female, in which a display of force and, doubtless, the sight of the blood of the vanquished, is pleasing to the female, and stimulates in her a reciprocal desire. Ascending in the scale of animal evolution we come to the tribal state of primitive man, and find that with certain barbarous tribes it became a vital necessity, from the scarcity and difficulty of obtaining food, and the necessity of swift migration, often, to escape hostile tribes, to sacrifice all girl babies and feeble adults to tribal safety. Out of this grew a scarcity of women and the necessity of capturing wives from other tribes (Exogenous Marriages—Spencer, *Principles of Sociology*, p. 622). In time it became a distinction to have a captured wife. It was a token of prowess, skill and bravery. A survival of the custom is found today in the practices amongst certain low forms of man; *e. g.* Spencer says: "A young Dyak must prove his bravery by bringing back the head of an enemy before he is permitted to marry. When the Apache warriors return unsuccessful, the women turn away from them with indifference and contempt. They are upbraided as cowards or for want of skill and courage, and are told that such men should not have wives." * * * "In a tribe containing many warriors who had wives taken from enemies, and who were regarded as more honorably married than the rest, there would result an ambition, if not to capture a wife, still to seem to capture a wife"; hence arose the mock capture of wives,—captured not from warriors of other tribes, but against the will of the woman and that of her parents in the same tribe. This form of capture is still practiced by barbarous people in Africa, in Australia, amongs the Sinai Arabs, the Fuegians, and other uncivilized countries. Hence it is an easy gradation to the purchase of a wife from her parents, generally prevalent amongst savage and semi-civilized people—and to that semblance of capture—a play of mock capture prevalent today among many people as a part of the marriage ceremony. We see the shadow of reflection of it even among civilized people, in the game of "tag" between girls and boys, in which the boy must chase the girl, and when caught, kiss her. By the bye, it is said by authorities cited, that the kiss is the survival of the love bite, still practiced amongst certain races, notably, the Esquimaux.

It is interesting and instructive to trace a custom back to its origin, as Spencer, Ellis, McLennan, Sir John Lubbock and others have done, in regard to the custom of mock capture of the bride as part of the marriage ceremony by many savages. It is not generally known, and I have the authority of Clifford Howard in his book, "Sex Worship," for saying, that the emblems of the worship of Phallus and Priapus survive today in the gothic architecture of our churches, the spire, the windows and doors—the sanctum sanctorum or innermost holy, being significant of that religion which worshipped the generative organs as the source and origin of all life. The plumes on the hearse today, Spencer suggests, is an echo of the custom originating in feudal times, of carrying the knight's plumed helmet,—his arms and trappings,—on his favorite steed, at the head of his funeral procession.

Sadism is a survival of marriage by capture—as practiced by primitive man and his brute progenitors. Or, rather, it is atavistic. This view is held by Kiernan (*Psychological Aspect of the Sexual Appetite. Alienist and Neurologist, April,* 1891), citing whom, Kraft-Ebing says:

"Kiernan, who has several authorities in Anglo-American literature, starts from the assumption of several naturalists (Dollinger, Drysdale, et al.) which conceive, the so-called conjunction—a sexual act, in certain low forms of animal life, to be cannibalism, a devouring of the partner in the act. He brings into immediate connection with this the well-known facts that at the time of sexual union crabs tear limbs from their bodies, and spiders bite off the heads of the males, and other sadistic acts performed by rutting animals with their consorts.* From this he passes to lust murder and other lustful acts of cruelty in man, and assumes that hunger and the sexual appetite are, in their origin, identical; that the sexual cannibalism of lower forms of animal life has an influence on the higher forms and in man, and that sadism is an example of avatism."

The American negro is only a few generations removed from savagery. Naturally the brute instinct is strong in him, and unrestrained by influences which keep it in abeyance in civilized countries, measurably, at least, it will express itself in those acts of which we have been speaking. In recent years it seems, as Burton

---

*The female of that familiar winged insect, the "Devil's Horse," (*Hymenoptera; mantis religiosa*) devours the male after the conjunction, and the queen bee flies away with the drone she has selected to fertilize her for the next generation of workers (*le vol nuptial*). After fecundation she rips open the abdomen of the male and serenely scatters his intestines to the wind.

has said of ancient times, that, even the fear of the fagot, to say nothing of other forms of capital punishment, is powerless to prevent those horrible lust murders of children by negroes. The cause, in my judgment, is sadism, a reversion to the type of a not remote ancestry. There are contributory causes to be presently mentioned. As a slave, the negro was in ignorance and complete subjection. The law recognized no rights on the part of a slave. The law and the lash kept him in subjection, while on plantations every facility for the gratification of his lust was afforded with women of his kind, for breeding slaves was a profitable business. Marriage amongst them was encouraged. There was no perversion of the sexual sense. The males did not desire the white women, nor dream of ravishing the white children. The whites to whom he belonged were immeasurably above and beyond him, and he no more dreamt of social equality with them than with the angels. Freed from these restraints, only partly educated, lazy, often idle and dissipated, frequenting low drinking dives and vile haunts of vice, corrupt and bad, allowed to associate with dissolute white women,—some of them dream of social rights and equality. He has been told by fanatics that he is as good as the white rebels to whom his parents belonged, and their descendants; race hatred is engendered, and despite torture and certain death staring him in the face, the rape fiend, the negro sadist, wreaks his vengeance and spite on some innocent child and gratifies, in that unnatural manner, his abominable lust. In how far the example set by the Chief Executive of the United States in socially recognizing the negro may have encouraged the rape fiend, and thus been a factor in the increase of the crime, it is hard to say; but it is not unreasonable to say that, doubtless it has given an impetus to both hatred of the whites and desire for revenge, the more so, perhaps, because the illustrious exemplar of negro equality is himself a grandson of a distinguished slave owning aristocrat of the Old South.

So much then, for the cause of rape by negroes.

We of the South are confronted with this horrible problem: There have been forcibly turned loose upon us some millions of blacks, who, however, for the most part, are orderly, industrious and well-behaved, and who are entitled to the respect and encouragement of all right-thinking people. There are amongst them, however, individuals in whom there exists that form of sexual perversion we have been describing, and other forms that lead to sexual crimes besides rape. I have endeavored to assign a cause for it; now, is there a remedy? The title of my paper is, "The Cause and

Prevention of Rape." I do not say that there is a remedy, or that it can be prevented or cured; but I do say that hanging and burning at the stake have signally failed to check the evil. Indeed, I venture the belief that by "suggestion," it has rather tended to increase it. Haslam, an English authority, says, that of 167 persons executed in England, all but one had witnessed an execution. The horrible execution by fire of the negro lust murderer, Henry Smith, at Paris, Texas, must have been known to every black in Texas, yet within a week there occurred other assaults by negroes on white girls only a little less revolting. Right here in Austin, shortly after the occurrence mentioned, a negro was hanged in the jail yard for rape of a 13-year-old white girl at Webberville. The physician who attended her testified to the horrible mutilation of the child. She survived, however, and under our laws, had to appear in court and exhibit her shame, humiliation and suffering. This is as disgraceful as it is absurd, and in itself constitutes some justification of lynching. The negroes were allowed to hold so-called religious services on the scaffold. The preacher told the assembled blacks and scattering whites that "Brother" Nichols (the culprit) had found forgiveness; that his soul had been washed white in the blood of the Lamb, and that he was going from this vale of tears, where the white man never ceases from troubling and the good darkey can not rest, straight to the bosom of God! The black cap was adjusted and he was launched into eternity to the tune of "Nearer My God to Thee," sung by a hundred black throats. This is no fancy sketch, but an actual occurrence right here at the Capital of a great State, the seat of the University, and the home of scores of Christian churches. Imagine the effect of such a scene and teaching upon the ignorant and superstitious negro. What does it "suggest?" Go thou and do likewise and go to glory! It is a premium on rape, a golden harp in the realms of the blest. Is such calculated to lessen the prevalence of this horrible crime?

Nor are the proposed remedies likely to be more effectual. I mean that proposed by John Temple Graves, colonizing the negro, separating the races. It is utterly impracticable and undesirable. The great majority of the negroes are inoffensive and are useful,—indispensable in the South as laborers. Justice Brewer's proposed remedy for rape and its consequences (lynching) is almost puerile. He proposes that in rape trials there shall be no appeal; but a swift execution, to satisfy the outraged people. Why have a trial? A judicial investigation is for the purpose of ascertaining if the accused is guilty, and, if guilty, to fix the penalty. In every case where there is the least doubt, either of the identity of the accused or of the crime, there should be an investigation, but where there is no doubt as to either, why subject the victim—most frequently a tender young girl—to the shame, mortification and suffering incidental thereto, and the State to the expense? It is a farce in those cases where the evidence is conclusive, *prima facie;* the death of the fiend has already been determined upon. Indeed, the lust murderer is outside the pale of the law. He has no rights that should be respected. He is a monster, a unit dangerous to society, and should be eliminated. The question of "responsibility," —granting that sadism is a form of insanity—cuts no figure. He should be killed—with as little ceremony as a community would put to death any other deadly animal—say a man-eating tiger, for instance, loose in their midst, or a rattle-snake. There should be no vengeance, no torturing, of course; no one would think of torturing a bull or dog or tiger, or snake; he should simply be killed, and the outraged community, for its own protection, should do it, without the farce of a trial, in cases in which there is no doubt of the crime and the identity of the criminal. In less horrible cases of sexual crimes—such as are recognized by jurisprudence and are evidence of sexual perversion, *exempli gratia,* sodomy, buggery, pederasty,— upon conviction before a court, the offender should be rendered forever incapable of a repetition of the act, and of reproducing his kind, in whom the taint would most probably survive. This should be done by a surgeon, upon order of the court, and there should be a law upon our statute books authorizing it. I do not mean simply the removal of the glands, for while that destroys the power of procreation, it in no degree lessens the libido or the power of erection and penetration. See Havelock Ellis on this subject. It would carry this paper beyond the limits of time at my disposal to enter upon a discussion of this phase of my subject. The remedy should consist of the ablation of the entire genital apparatus, for, as stated, castration does not remove the impulse nor the power, and Ellis says that "certain Roman ladies greatly affect this style of eunuch for obvious reasons; *vult futui Gallia, non parere.*

Our esteemed fellow citizen, Judge A. W. Terrell, ex-minister to Turkey, says that when the Arab slave dealers are returning from Africa with slaves they have bought or stolen or captured,—for the Constantinople market, they camp by a river where there is a sandbar. Holes are dug in the wet sand into which the males are buried up to the chin, after having the entire external genitalia removed with one sweep of a sharp knife. Wet sand is then packed down firmly around the wretch, and if he does not bleed to death, as they frequently do, when he is taken out (in about a week) he is a true eunuch worth $500.

Seeing that prevalent methods of dealing with rapists are unavailing; that proposed remedies are impracticable or likely to be useless, I really think it would be "worth while" to try asexualization in all cases of attempted rape where there has been no penetration, and in all established cases of sexual perversion which has been expressed in unnatural acts of gratification. For the successful rapist,—white or black,—and for the rape-murderer, death, swift and without pity or remorse. The preservation of self and the protection of home are man's highest instincts and first duty.

I really believe that this measure will do more to check the evil of criminal assault than anything else. A negro values the "witnesses of manhood" more than life, and would give up life with less reluctance. The fear of the loss of those possessions would have more terror and more restraining influence on his acquaintances, and on the evil disposed—and the moral effect would be greater than that of shooting, hanging, or death at the stake. It is the only rational remedy. It is based on science, and will be at once, punitive, curative and preventive,—perhaps.

